# Supplementary material for: Resolvin T4 enhances macrophage cholesterol efflux to reduce vascular disease
Source: Nat Commun. 2024 Feb 5;15:975. doi: 10.1038/s41467-024-44868-1 (PMC10844649; doi:10.1038/s41467-024-44868-1)
Supplement: Supplementary file 1 — Supplementary Information [file 41467_2024_44868_MOESM1_ESM.pdf]

Resolvin T4 enhances macrophage cholesterol efflux to reduce vascular disease

Mary E. Walker[1*], Roberta De Matteis[1*], Mauro Perretti[1,2] and Jesmond Dalli[1,2]

[1] William Harvey Research Institute, Barts and The London, Faculty of Medicine and Dentistry, Queen Mary University of London, Charterhouse Square, London, EC1M 6BQ UK.

[2] Centre for Inflammation and Therapeutic Innovation, Queen Mary University of London, London, UK.

*These authors contributed equally

[+]Corresponding author: Prof Jesmond Dalli Ph.D, William Harvey Research Institute, John Vane Science Centre, Charterhouse Square, London. EC1M 6BQ. E-mail: j.dalli@qmul.ac.uk, Tel: +44 (0) 207 882 8263

Supplementary figures

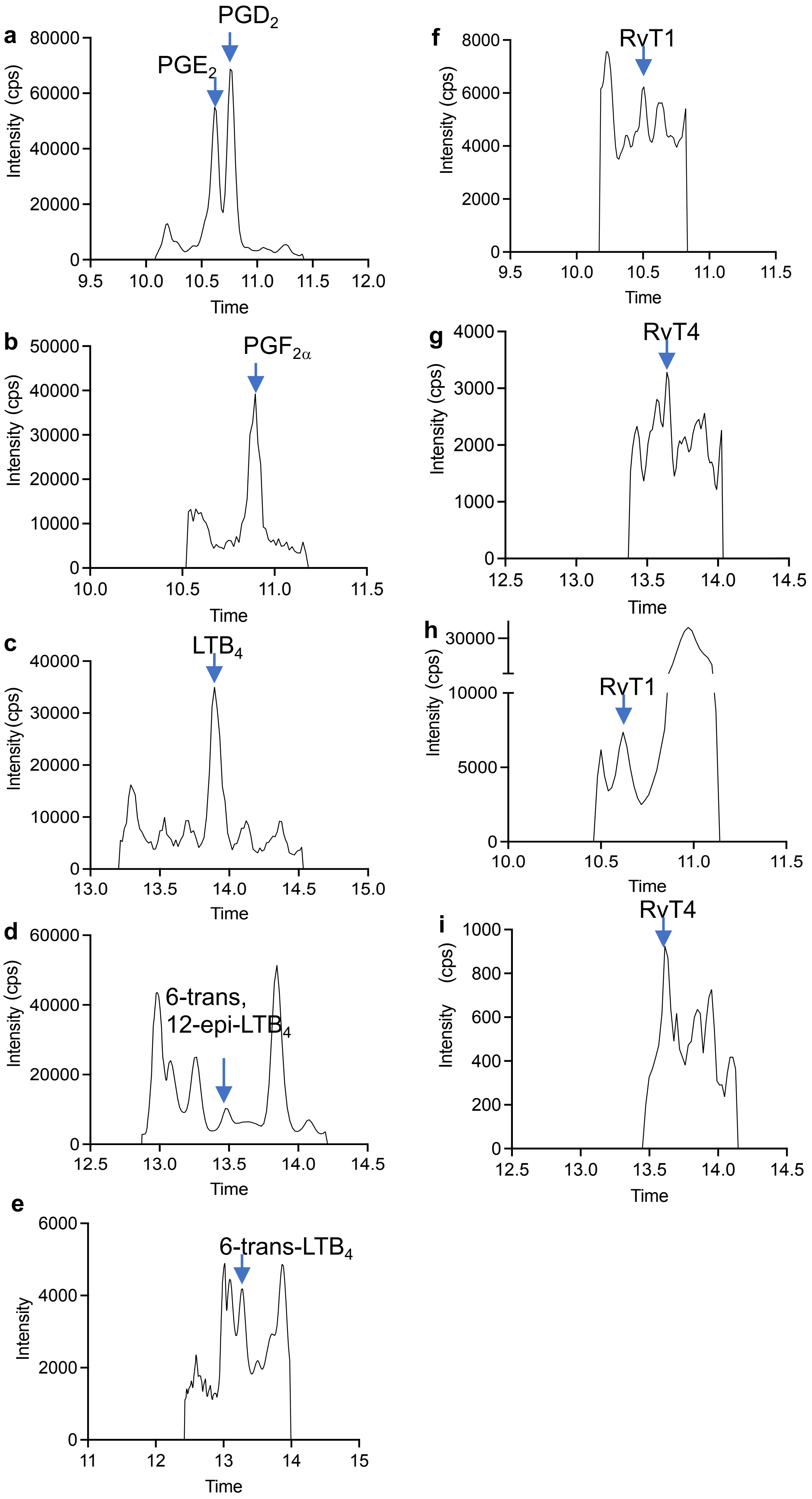

Supplementary Figure 1. Representative chromatograms employed in the identification of lipid mediators in plasma and aortas.

Wild-type C57BL/6 mice were fed chow or Western-style diet. K/BxN serum-induced arthritis was initiated at 11 weeks and sustained by giving three weekly boosters (See Figure 1A). (A-E) Aortas were isolated (A,B) prostaglandins and (C-E) leukotrienes were identified in aortic sections using targeted LC-MC/MC profiling. Figure illustrates replotted representative chromatograms employed for the identification of lipid mediators in aortas. Arrows denote the retention time for each of the lipid mediators. (F,G) Wild-type C57BL/6 mice were fed chow or Western-style diet. K/BxN serum-induced arthritis was initiated at 11 weeks. Six days later plasma was collected. (F) RvT1 and (G) RvT4 were identified using targeted LC-MC/MC profiling. Figure illustrates replotted representative chromatograms employed for the identification of lipid mediators in aortas. Arrows denote the retention time for each of the lipid mediators. (H-I) Arthritic WT and ApoE$^{-/-}$ mice were fed a standard chow diet or WD for 11 weeks (see Figure 1A). Plasma RvT1 and RvT4 were identified using LC-MS/MS profiling. Figure illustrates replotted representative chromatograms employed in the identification of (H) RvT1 and (I) RvT4 in aortas. Arrows denote the retention time for each of the lipid mediators.

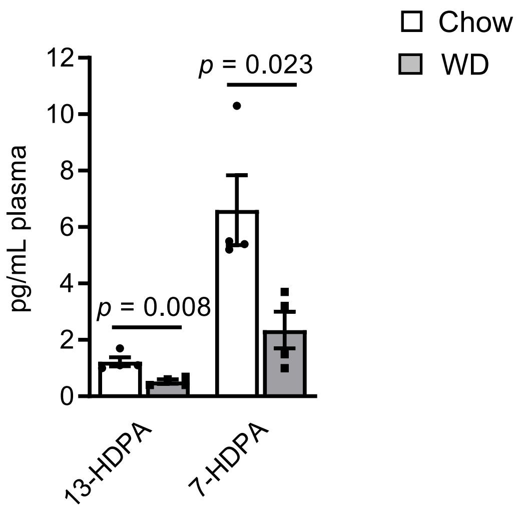

Supplementary Figure 2. Decreased levels of RvT pathway markers in mice fed a Western-style diet.

Wild-type mice were fed chow or WD for 6 weeks and inflammatory arthritis was initiated *via* the administration of arthritogenic K/BxN serum (100µl per mouse, *i.p*). Plasma was collected and pathway markers for COX-2 (13-HDPA) and ALOX5 (7-HDPA) activity were assessed using lipid mediator profiling. Error bars, mean ± s.e.m.; n = 3 per group; *p* value was calculated using Student's t-test.

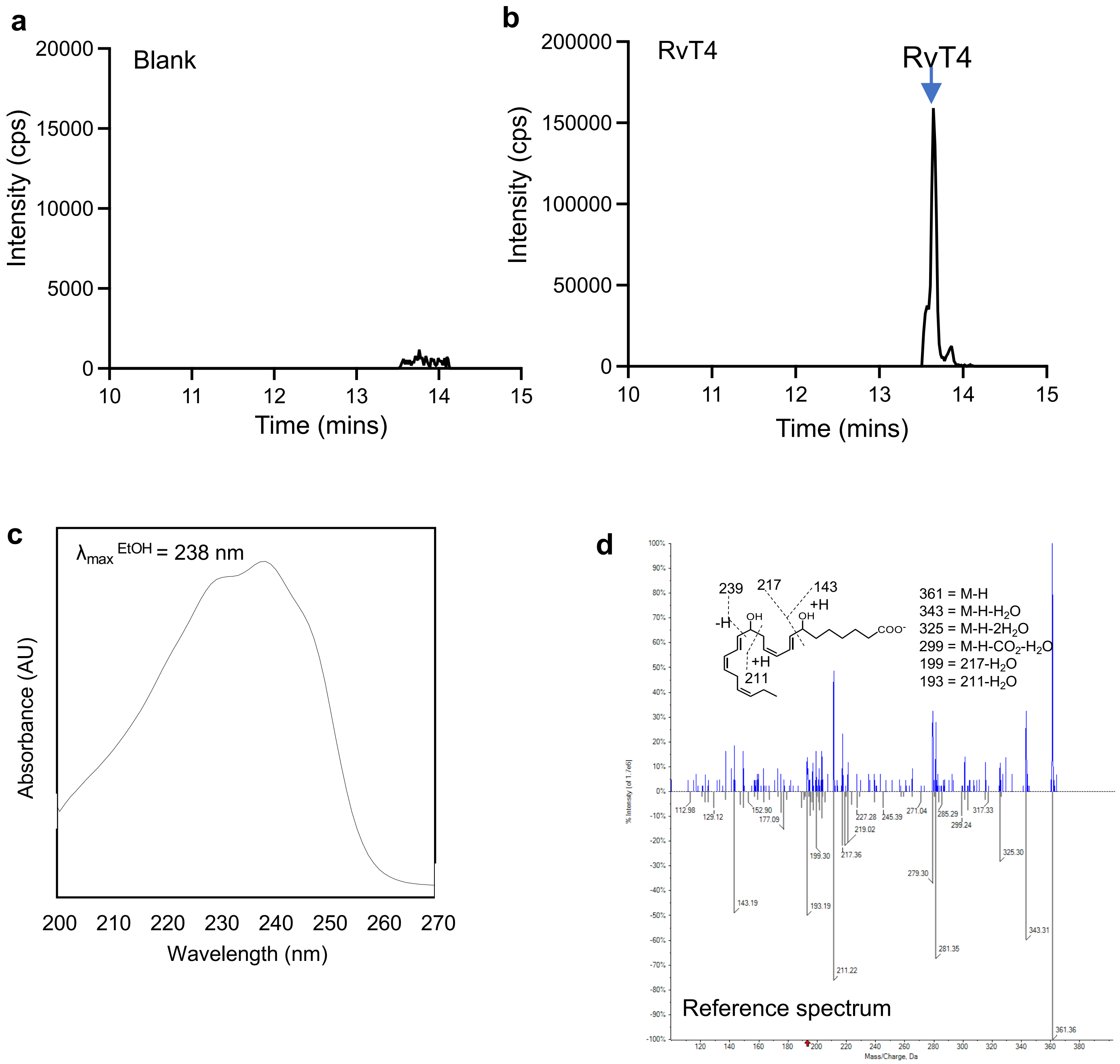

Supplementary Figure 3. Characterization of RvT4 produced via biogenic synthesis.

RvT4 was produced by incubating 13R-HDPA with potato 5-lipoxygenase, and isolated using RP-HPLC (see methods for details). **(A,B)** Representative MRM chromatogram (361 > 211) of **(A)** blank control **(B)** biogenic RvT4. **(C)** Characteristic UV chromophore for RvT4. **(D)** Biogenic RvT4 (top) and reference RvT4 (bottom) MS/MS spectra employed in the identification of RvT4 (inset, diagnostic ions; M, molecular mass).

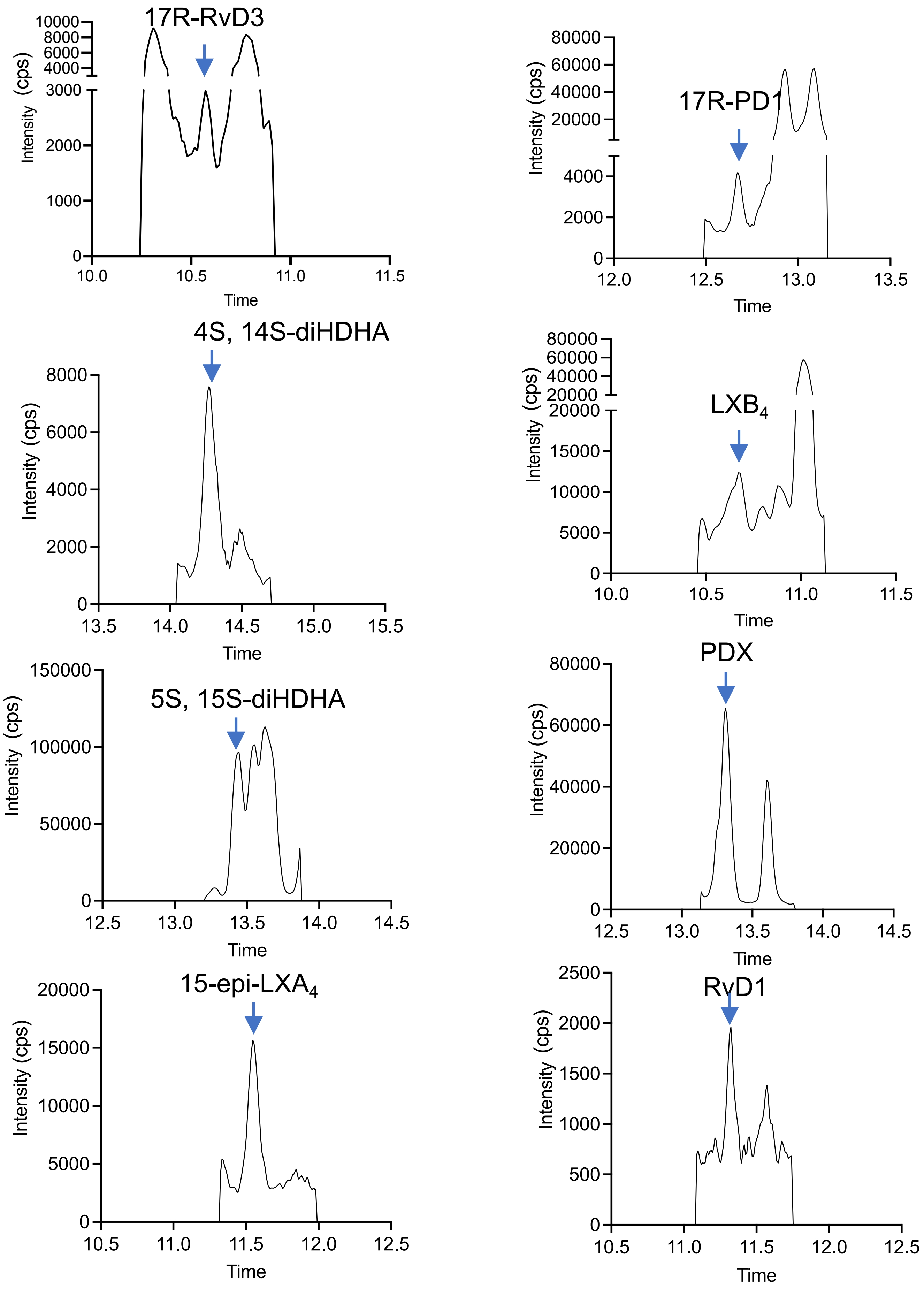

17R-RvD3
Intensity (cps)
10000
8000
6000
4000
3000
2000
1000
0
10.0
10.5
11.0
11.5
Time
17R-PD1
Intensity (cps)
80000
60000
40000
20000
4000
2000
0
12.0
12.5
13.0
13.5
Time
4S, 14S-diHDHA
Intensity (cps)
8000
6000
4000
2000
0
13.5
14.0
14.5
15.0
15.5
Time
LXB_4
Intensity (cps)
80000
60000
40000
20000
20000
15000
10000
5000
0
10.0
10.5
11.0
11.5
Time
5S, 15S-diHDHA
Intensity (cps)
150000
100000
50000
0
12.5
13.0
13.5
14.0
14.5
Time
PDX
Intensity (cps)
80000
60000
40000
20000
0
12.5
13.0
13.5
14.0
14.5
Time
15-epi-LXA_4
Intensity (cps)
20000
15000
10000
5000
0
10.5
11.0
11.5
12.0
12.5
Time
RvD1
Intensity (cps)
2500
2000
1500
1000
500
0
10.5
11.0
11.5
12.0
12.5
Time

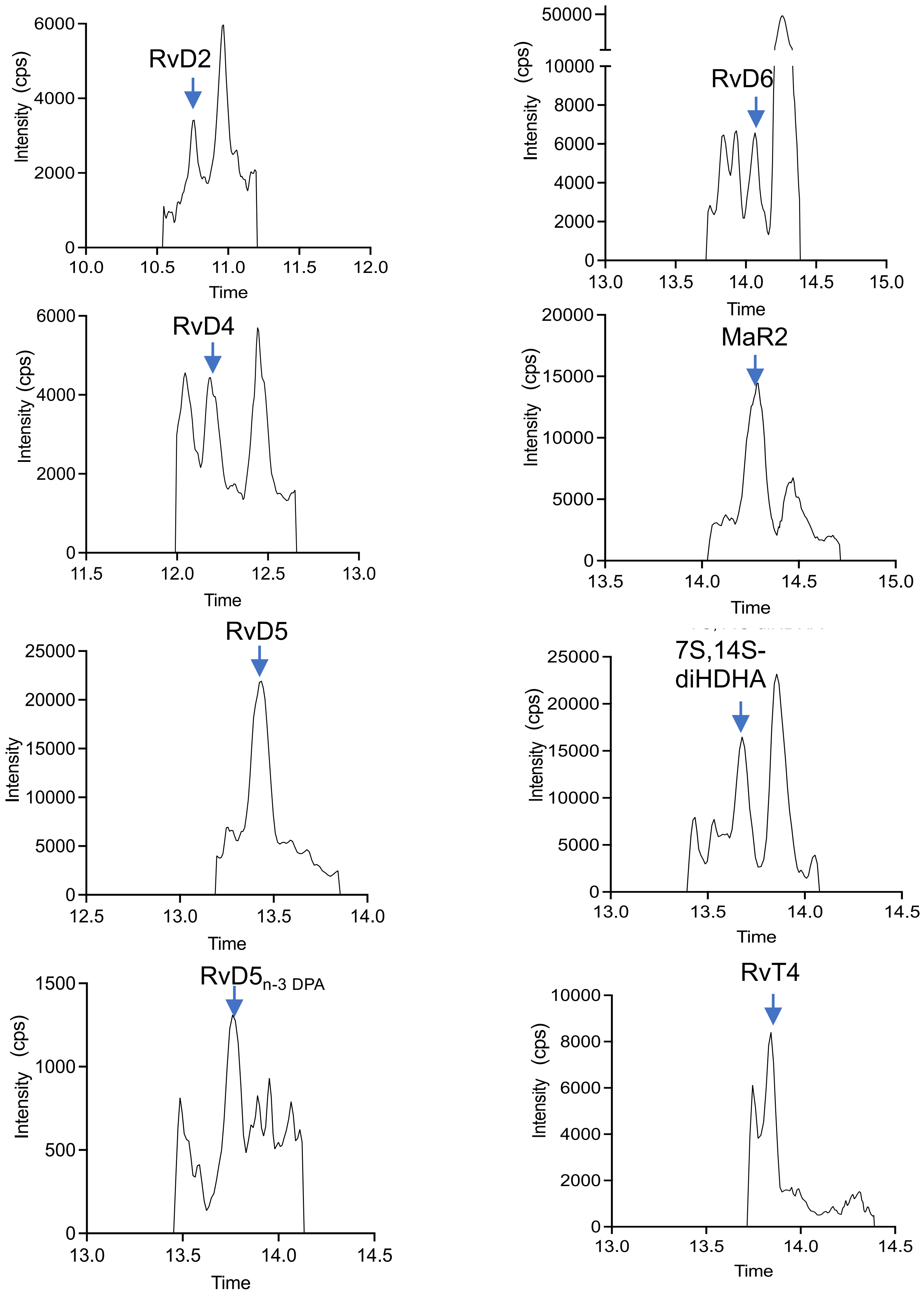

Supplementary Figure 4: Representative chromatograms for identified SPM in aortas.

Wild-type C57BL/6 mice were fed a Western-style diet and K/BxN serum-induced arthritis sustained for 4 weeks. After the final boost, mice were injected with vehicle or 75 ng RvT4 on alternating days for 10 days. Mice were culled at 16 weeks and aortas collected for lipid mediator profiling. Figure denotes replotted representative chromatograms employed for the identification of lipid mediators in aortas. Arrows denote the retention time for each of the lipid mediators.

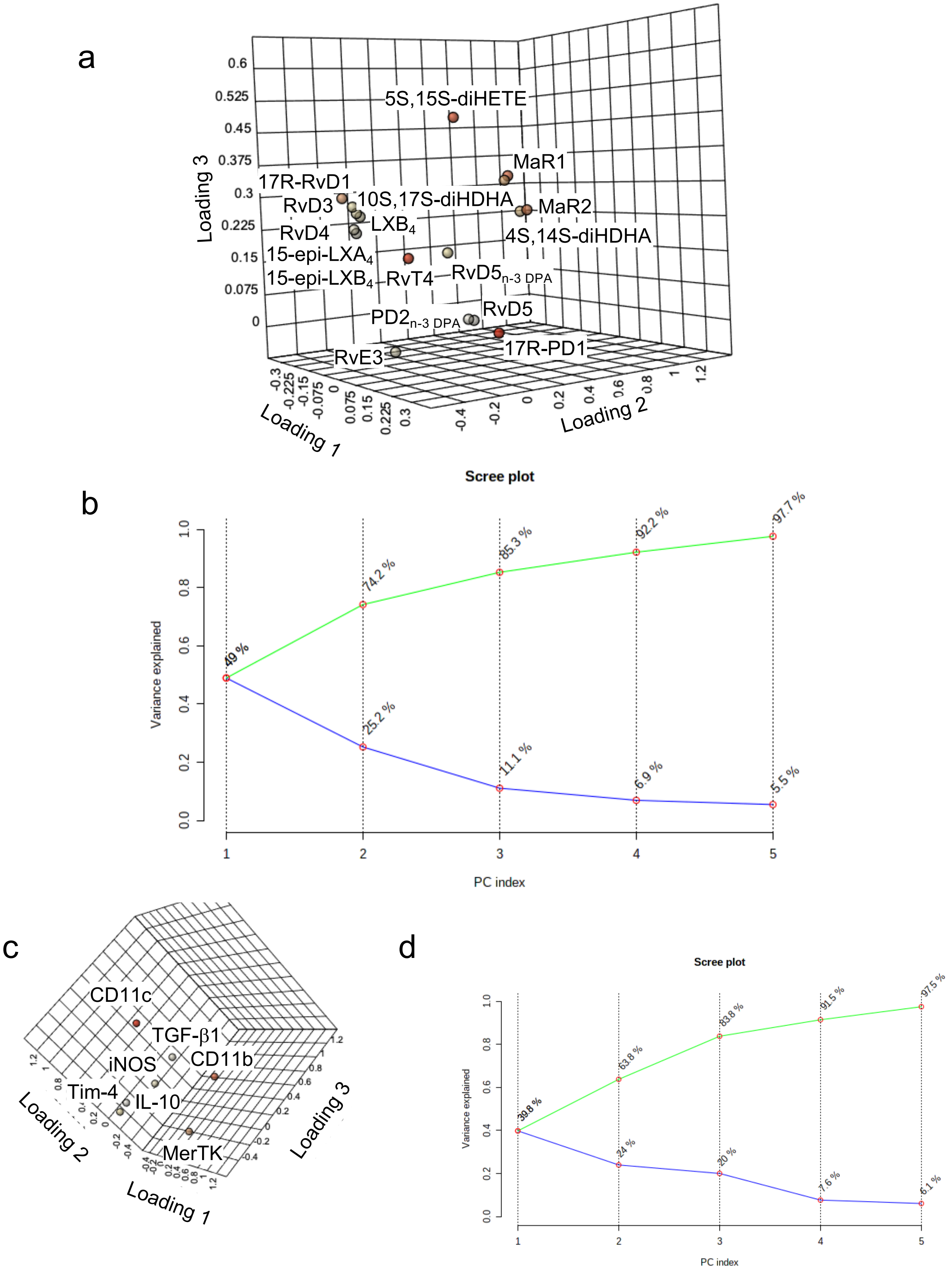

Supplementary Figure 5. PCA Loading Plots and Scree Plots for aortic lipid mediator profiles and macrophage phenotypic marker expression.

Wild-type C57BL/6 mice were fed a Western-style diet and K/BxN serum-induced arthritis sustained for 4 weeks. After the final boost, mice were injected with vehicle or 75 ng RvT4 on alternating days for 10 days. Mice were culled at 16 weeks and aortas collected for (A,B) lipid mediator profiling, which was assessed using PCA with (A) being the loading plot for data reported in Figure 2D and (B) the associated Scree plot. (C,D) Expression of macrophage phenotypic markers was evaluated using flow cytometry (C) being the loading plot for data reported in Figure 2F and (B) the associated Scree plot.

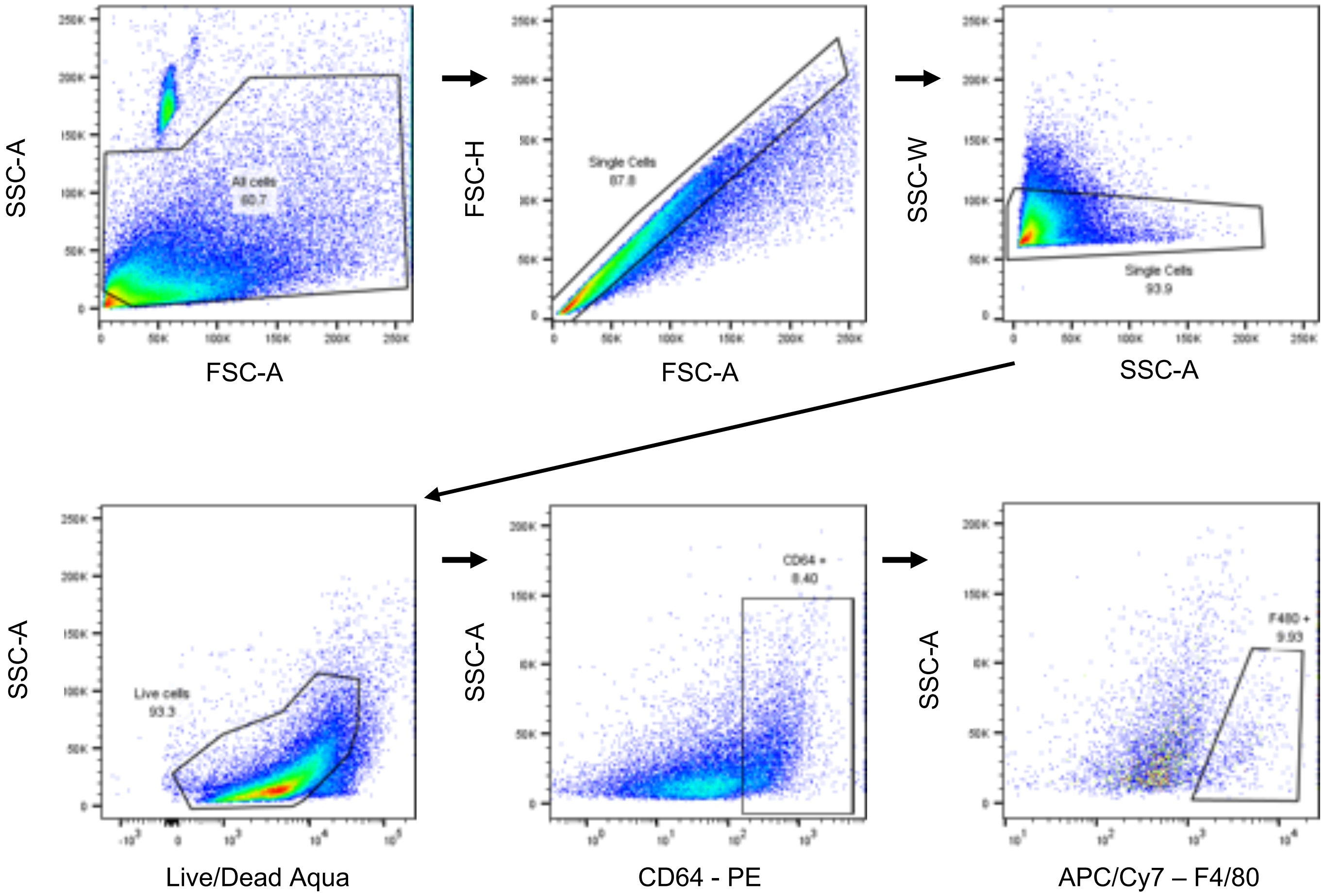

Supplementary Figure 6. Flow cytometry gating strategy for characterization of aortic macrophages.

Wild-type mice were fed WD for 7 weeks and inflammatory arthritis was initiated *via* the administration of arthritogenic K/BxN serum (100µl per mouse, i.p). After 6 days aortas were harvested and incubated for 16 hours with vehicle (0.03% ethanol) or 1 nM RvT4. Single-cell suspensions were prepared, and macrophage markers were identified and quantified using fluorescently labelled antibodies and flow cytometry. To identify cells of interest, debris was excluded using FSC/SSC gating around cell populations. Doublets were excluded using FSC-A/FSC-H gating around single cell population, followed by SSC-A/SSC-W. Live cells were identified as Live/Dead Aqua low, and macrophages were identified as CD64+ and F4/80+ cells.

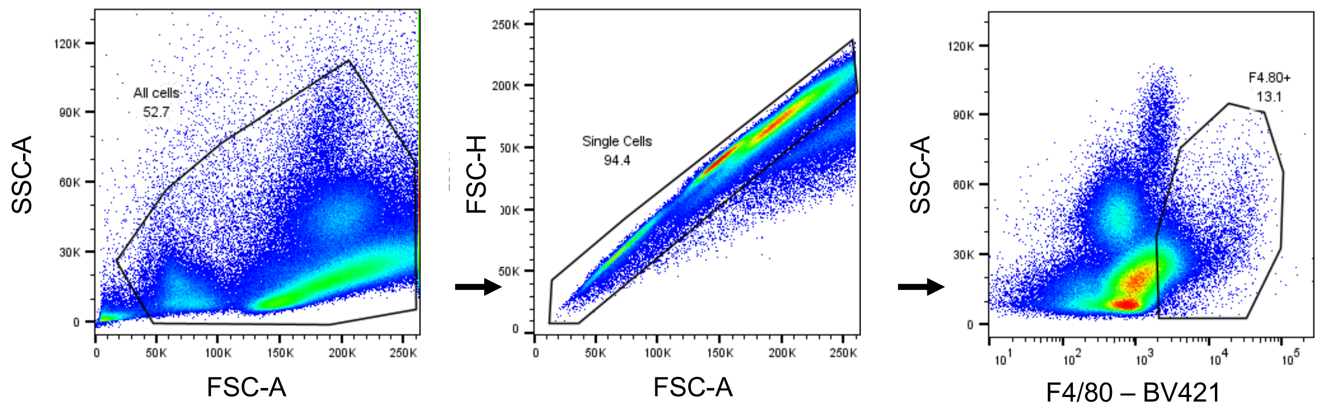

Supplementary Figure 7. Flow cytometry gating strategy for measuring *in vivo* cholesterol efflux in peritoneal macrophages.

ApoE$^{-/-}$ mice were injected i.p. with 5 μg BODIPY-FL-cholesterol in PBS. After 16 hours, peritoneal lavages were carried out. To identify cells of interest, debris was excluded using FSC/SSC gating around cell populations. Doublets were excluded using FSC-A/FSC-H gating around the single cell population. Cells positive for F4/80 were selected, and BODIPY-FL fluorescence intensity was measured within this population of cells.

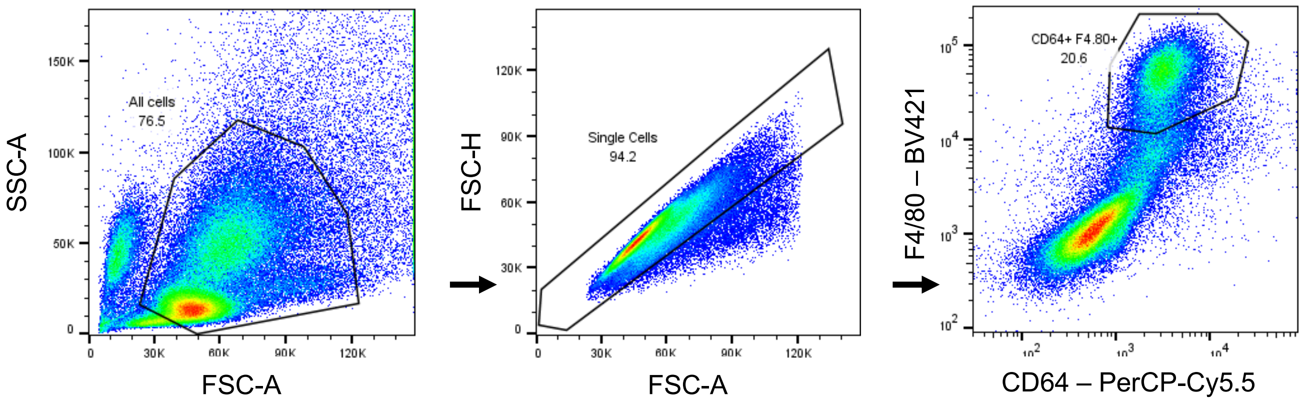

Supplementary Figure 8. Flow cytometry gating strategy for characterization of peritoneal macrophages.

ApoE$^{-/-}$ mice were injected with vehicle, 75 ng or 150 ng RvT4 and then PKH26-labelled apoptotic HL60 cells. After 60 minutes, lavage fluid was collected. Macrophage markers were identified and quantified using fluorescently labelled antibodies and flow cytometry. To identify cells of interest, debris was excluded using FSC/SSC gating around cell populations. Doublets were excluded using FSC-A/FSC-H gating around the single cell population. Cells positive for CD64 and F4/80 were then selected.

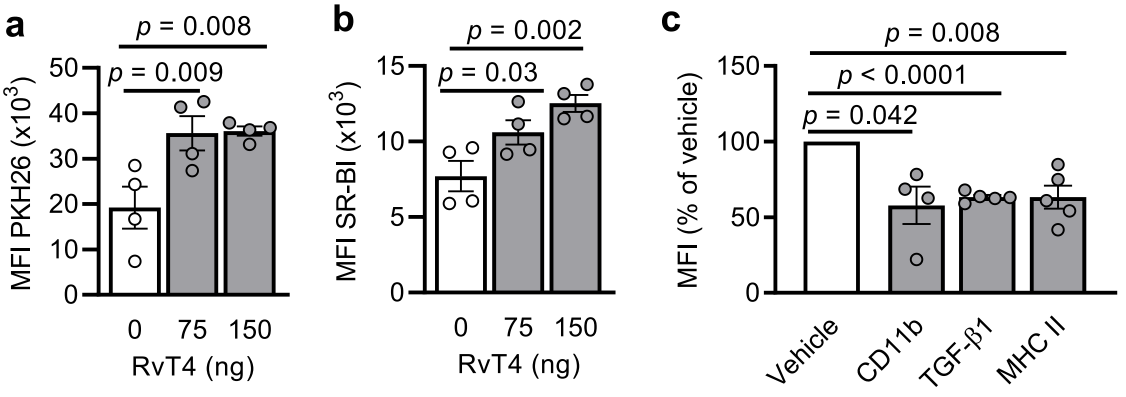

Supplementary Figure 9. RvT4 regulate macrophage phenotype, function and plasma membrane SR-BI expression in macrophages from *ApoE*$^{-/-}$ mice.

ApoE$^{-/-}$ mice were injected with vehicle, 75 ng or 150 ng RvT4 and then PKH26-labelled apoptotic HL60 cells. After 60 minutes, lavage fluid was collected. (A) Efferocytosis and (B) SR-BI surface expression were assessed on CD64+ F4/80+ cells using flow cytometry. n = 4 per group; *p* value was calculated using 1-way ANOVA. (C) Aortas of ApoE$^{-/-}$ mice fed a Western-style diet for 8 weeks were isolated and incubated with vehicle or RvT4. Single-cell suspensions were prepared from the remaining tissue and markers assessed on CD64+ cells using flow cytometry. n = 4 per group; Statistical differences were evaluated using one-sample t-test. Results are from two distinct experiments.

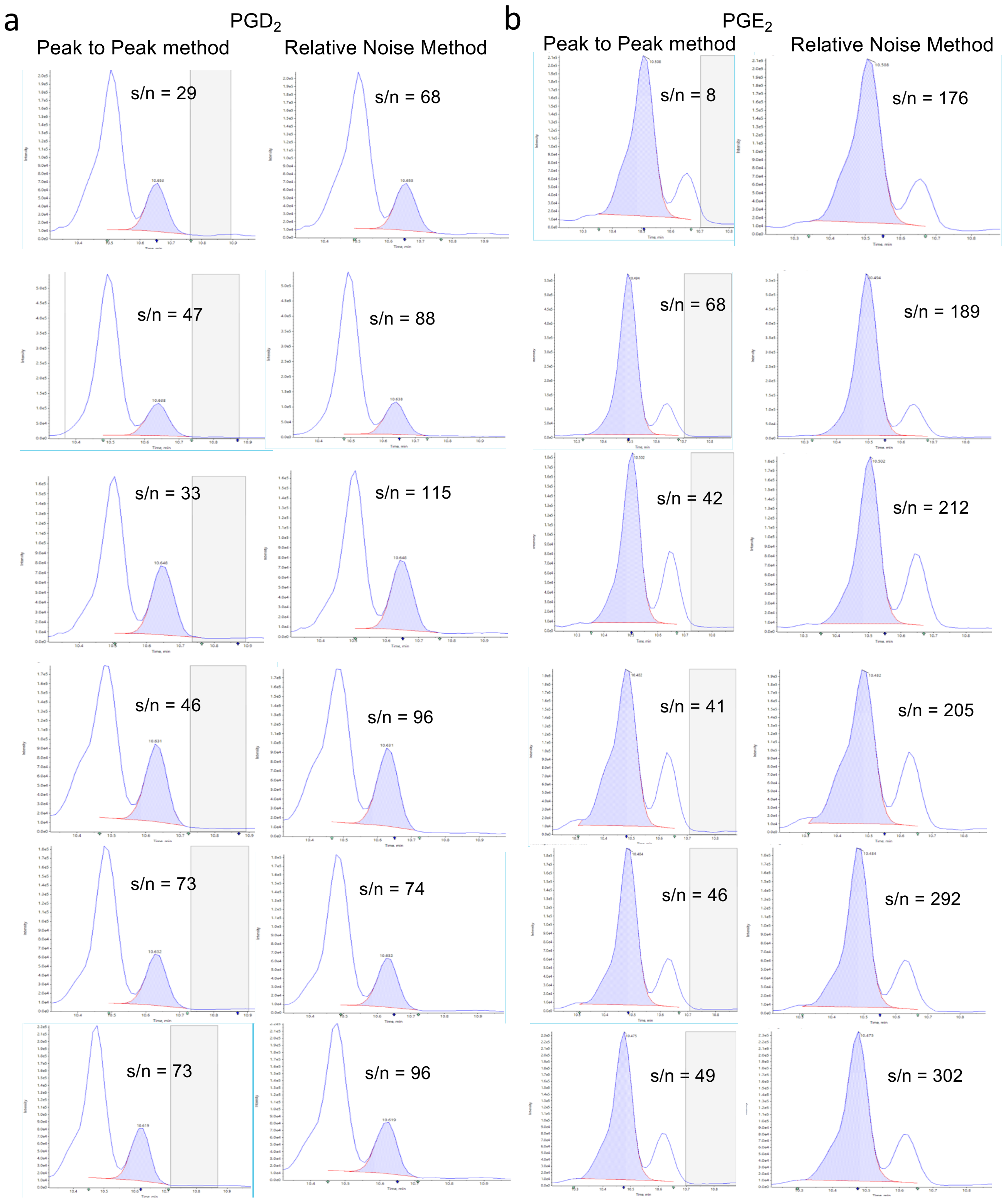

a
PGD2
Peak to Peak method
Relative Noise Method
s/n = 29
s/n = 68
s/n = 47
s/n = 88
s/n = 33
s/n = 115
s/n = 46
s/n = 96
s/n = 73
s/n = 74
s/n = 73
s/n = 96
b
PGE2
Peak to Peak method
Relative Noise Method
s/n = 8
s/n = 176
s/n = 68
s/n = 189
s/n = 42
s/n = 212
s/n = 41
s/n = 205
s/n = 46
s/n = 292
s/n = 49
s/n = 302

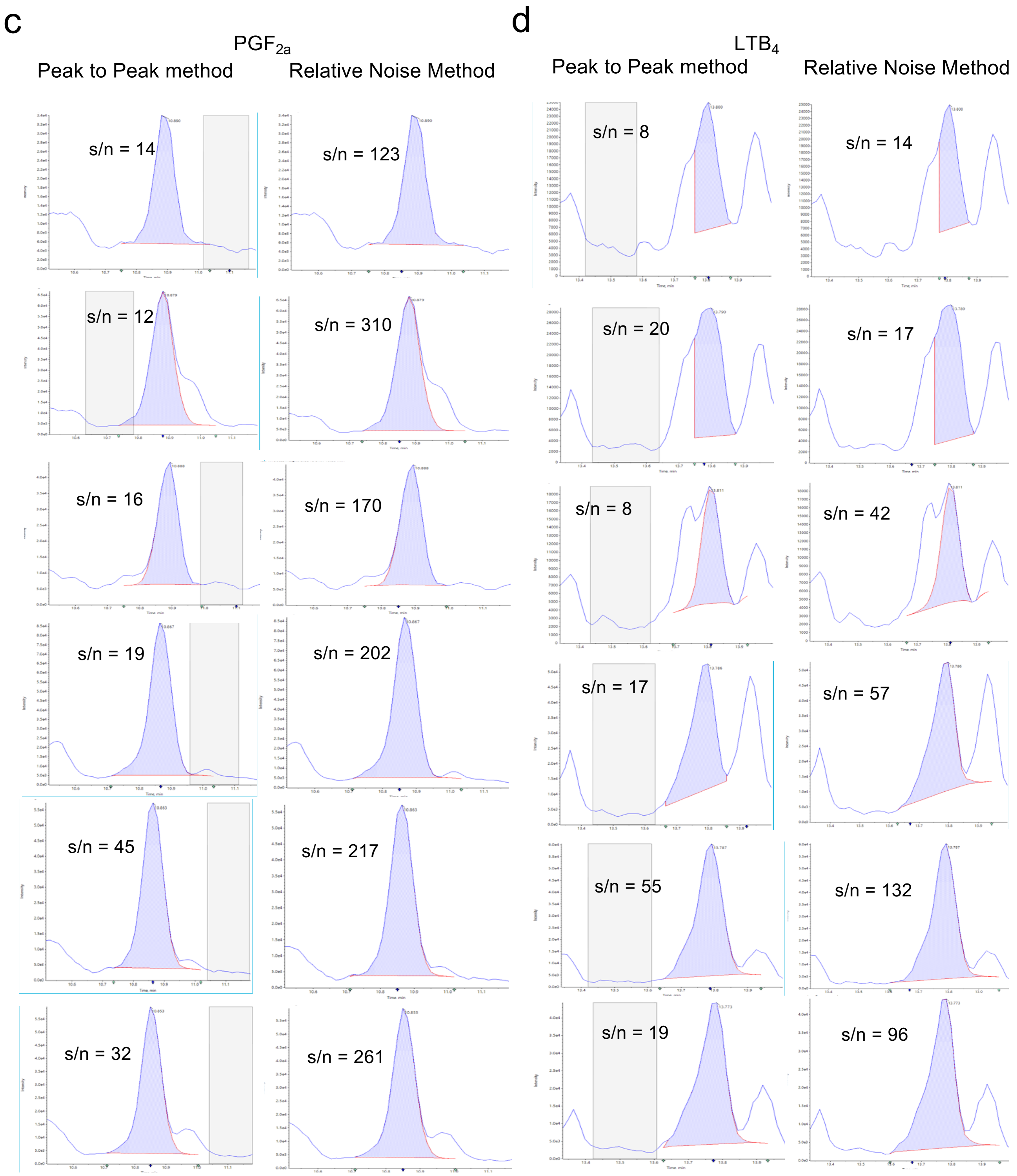

c
PGF2a
Peak to Peak method
Relative Noise Method
s/n = 14
s/n = 123
s/n = 12
s/n = 310
s/n = 16
s/n = 170
s/n = 19
s/n = 202
s/n = 45
s/n = 217
s/n = 32
s/n = 261
d
LTB4
Peak to Peak method
Relative Noise Method
s/n = 8
s/n = 14
s/n = 20
s/n = 17
s/n = 8
s/n = 42
s/n = 17
s/n = 57
s/n = 55
s/n = 132
s/n = 19
s/n = 96

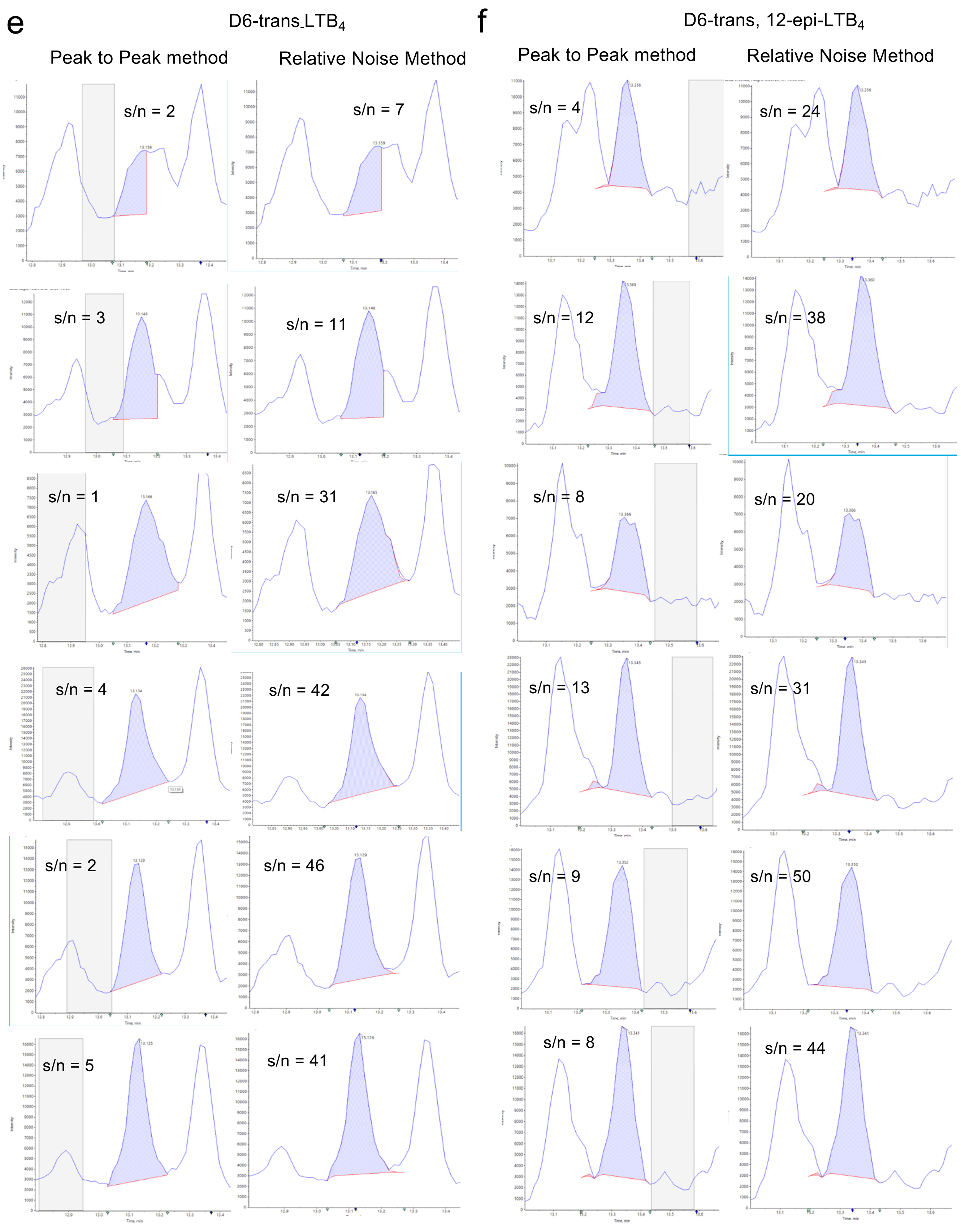

e
D6-trans_LTB4
Peak to Peak method
Relative Noise Method
s/n = 2
s/n = 7
s/n = 3
s/n = 11
s/n = 1
s/n = 31
s/n = 4
s/n = 42
s/n = 2
s/n = 46
s/n = 5
s/n = 41
f
D6-trans, 12-epi-LTB4
Peak to Peak method
Relative Noise Method
s/n = 4
s/n = 24
s/n = 12
s/n = 38
s/n = 8
s/n = 20
s/n = 13
s/n = 31
s/n = 9
s/n = 50
s/n = 8
s/n = 44

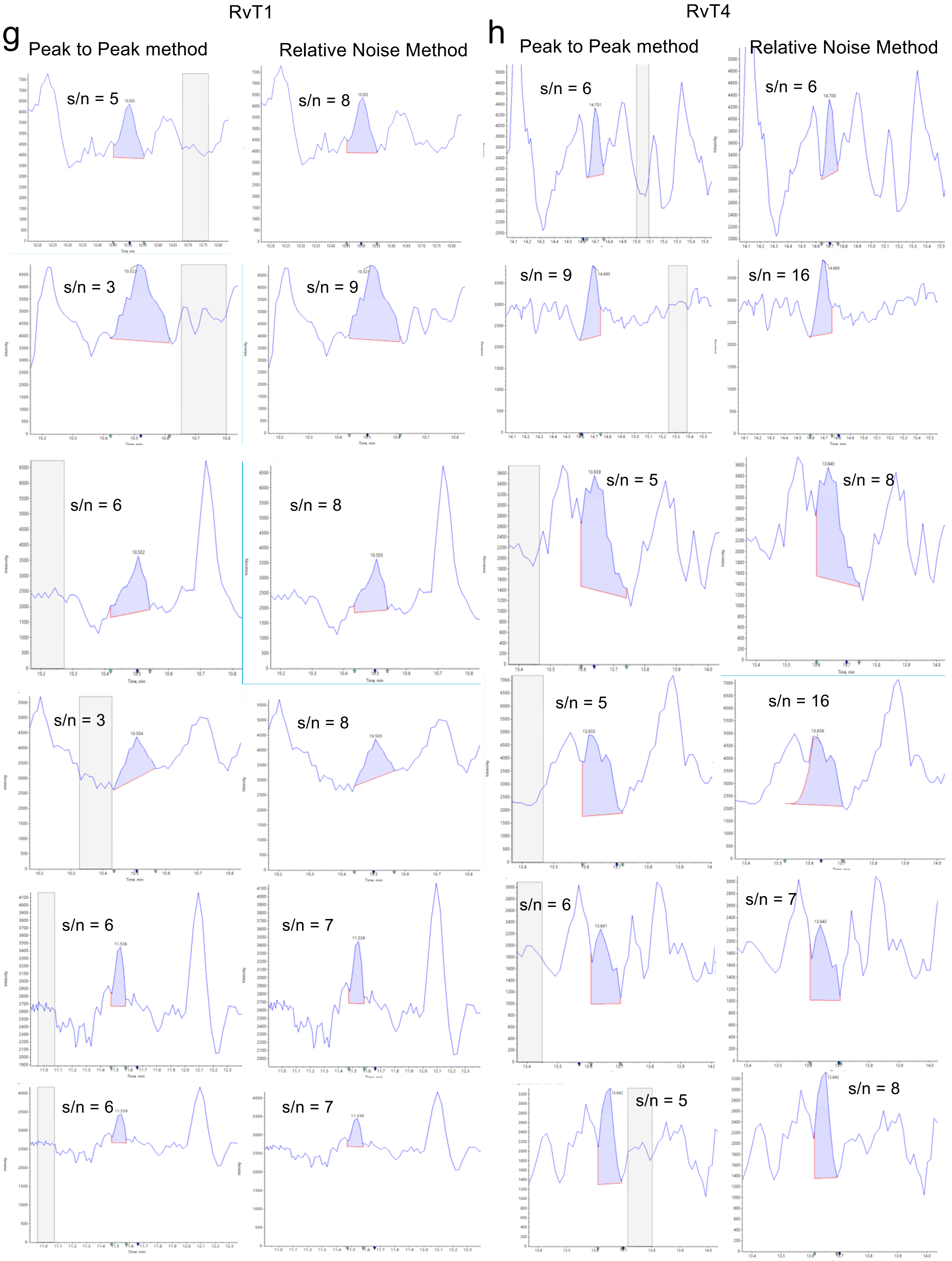

Supplementary Figure 10: Comparison of signal to noise ratios using relative noise and Peak to Peak methodologies. Screenshots of raw chromatograms for data presented in (A-F) Figure 1D and (G,H) Figure 1E. Regions highlighted in grey denote regions employed as 'noise' within the chromatogram. Regions denoted in blue are the integrated peaks.

Supplementary Tables

DHA-derived SPM	Vehicle Average	±	s.e.m	RvT4 Average	±	s.e.m
RvD1		-			-	
17R-RvD1	1.81	±	1.60	1.38	±	1.33
RvD2		-			-	
RvD3	0.75	±	0.37	1.13	±	0.53
17R-RvD3		-			-	
RvD4	4.36	±	3.62	3.88	±	2.02
RvD5	0.94	±	0.22	0.77	±	0.39
RvD6		-			-	
PD1		-			-	
PDX	2.64	±	0.67	4.74	±	3.37
17R-PD1	0.10	±	0.07	0.06	±	0.04
MaR1	0.82	±	0.25	2.09	±	1.86
MaR2	1.69	±	0.66	2.93	±	2.68
7S,14-diHDHA		-			-	
4S,14S-diHDHA	1.92	±	0.64	3.67	±	3.46
**n-3 DPA-derived mediators**						
RvT1		-			-	
RvT2		-			-	
RvT3		-			-	
RvT4	0.58	±	0.33	0.90	±	0.46
$RvD1_{n\text{-}3\ DPA}$		-			-	
$RvD2_{n\text{-}3\ DPA}$		-			-	
$RvD5_{n\text{-}3\ DPA}$	0.36	±	0.13	0.44	±	0.25
$PD1_{n\text{-}3\ DPA}$		-			-	
$PD2_{n\text{-}3\ DPA}$	1.24	±	0.48	1.06	±	0.70
10S,17S-diHDPA		-			-	
**EPA-derived SPM**						
RvE1		-			-	
RvE2		-			-	
RvE3	0.40	±	0.17	0.59	±	0.34
**AA-derived SPM**						
LXA_4	0.05	±	0.03	0.07	±	0.05
LXB_4	50.26	±	42.55	60.93	±	33.76
5S,15S-diHETE	36.75	±	11.28	57.85	±	38.29
15-epi-LXA_4	30.79	±	27.25	18.35	±	10.14
15-epi-LXB_4	133.82	±	123.67	80.18	±	49.56

Supplementary Table 1: Wild-type C57BL/6 mice were fed WD for 11 weeks. Five weeks after the initiation of WD inflammatory arthritis was initiated and sustained by the administration of arthritogenic K/BxN serum (100µl per mouse, *i.p.* weekly). 4 weeks after the onset of arthritis mice were administered vehicle or 75 ng RvT4 via *i.v.* injection on alternate days for 10 days. Descending aortas were harvested and placed in ice-cold methanol containing deuterated internal standards. Lipid mediators were extracted, identified, and quantified using lipid mediator profiling. Results are mean ± s.e.m. and expressed as pg / 10 mg tissue. n = 4-5 mice per group. - = below limits of the assay.

Supplementary Table 2: RvT4 alters aortic macrophage phenotype

**Marker** CD64+ F4/80+ cells	**Vehicle** (MFI)			**RvT4** (MFI)		
MerTK	7772.3	±	1143.7	14484.3	±	5691.8
CD11c	7878.3	±	2880.8	5570.8	±	1963.4
iNOS	5603.8	±	1209.6	4030.3	±	1424.2
MHC II	8796.3	±	5365.5	9438.3	±	4328.7
TIM-4	642.8	±	557.0	167.9	±	144.2
TGF-β1	593.3	±	172.8	210.3	±	139.6
CD11b	1174.0	±	207.8	943.8	±	72.9
IL-10	525.0	±	144.0	629.5	±	188.3

Wild-type mice were fed WD for 7 weeks and inflammatory arthritis was initiated *via* the administration of arthritogenic K/BxN serum (100µl per mouse, *i.p*). After 6 days aortas we harvested and incubated for 16 hours with vehicle (0.03% ethanol) or 1 nM RvT4. Single-cell suspensions were prepared, and macrophage markers were identified and quantified using fluorescently labelled antibodies and flow cytometry. Results are mean ± s.e.m. and n = 4 mice per group.

Supplementary Table 3: RvT4 regulates plasma lipid concentrations through macrophage SR-B1

	NTC and vehicle			NTC and RvT4			SR-BI KD and vehicle			SR-BI KD and RvT4		
**(mg/dL)**	**mean**	**±**	**s.e.m.**	**mean**	**±**	**s.e.m.**	**mean**	**±**	**s.e.m.**	**mean**	**±**	**s.e.m.**
**HDL cholesterol**	24.3	±	4.2	22.9	±	2.2	24.7	±	3.3	21.9	±	3.6
**LDL cholesterol**	949.9	±	163.9	798.5	±	108.6	848.5	±	88.9	770.8	±	118.7
**Total cholesterol**	974.2	±	160.9	821.4	±	110.1	873.2	±	90.8	792.7	±	120.9
**Triglyceride**	52.3	±	13.8	33.4	±	12.4	36.0	±	10.1	29.5	±	3.9
**Phospholipid**	290.6	±	37.7	160.8	±	18.3	192.6	±	24.5	211.7	±	20.9

ApoE$^{-/-}$ mice were fed a Western-style diet, and K/BxN serum-induced arthritis was initiated and sustained for 4 weeks. Over a 12-day period after the final K/BxN injection, mice were treated with *in vivo*-JetPEI-Man complexed with shRNA against Scarb1 or a non-targeting control (NTC), then with 75 ng RvT4 or vehicle (see schematic representation in Figure 7D). Plasma was collected and cholesterol, triglyceride, and phospholipid quantified.

Supplementary Table 4: Deuterium labels employed for the identification and quantification of lipid mediators

Mediator	Deuterium label employed to estimate recovery and retention time
RvD1	d_5-RvD2
17R-RvD1	d_5-RvD2
RvD2	d_5-RvD2
RvD3	d_5-RvD2
17R-RvD3	d_5-RvD2
RvD4	d_5-RvD2
RvD5	d_4-LTB_4
RvD6	d_4-LTB_4
PD1	d_4-LTB_4
PDX	d_4-LTB_4
17R-PD1	d_4-LTB_4
MaR1	d_4-LTB_4
7S, 14S-diHDHA	d_4-LTB_4
MaR2	d_4-LTB_4
4S,14S-diHDHA	d_4-LTB_4
RvT1	d_5-RvD2
RvT2	d_5-RvD2
RvT3	d_5-RvD2
RvT4	d_4-LTB_4
$RvD1_{n-3\ DPA}$	d_5-RvD2
$RvD2_{n-3\ DPA}$	d_5-RvD2
$RvD5_{n-3\ DPA}$	d_4-LTB_4
LXA_4	d_5-LXA_4
LXB_4	d_5-LXA_4
5S, 15S-diHETE	d_4-LTB_4
15-epi-LXA_4	d_5-LXA_4
15-epi-LXB_4	d_5-LXA_4
LTB_4	d_4-LTB_4
Δ6-trans-LTB_4	d_4-LTB_4
Δ6-trans, 12-epi-LTB_4	d_4-LTB_4
PGD_2	d_4-PGE_2
PGE_2	d_4-PGE_2
PGF_{2a}	d_4-PGE_2
