# Supplementary material for: Resolvin T4 enhances macrophage cholesterol efflux to reduce vascular disease
Source: Nat Commun. 2024 Feb 5;15:975. doi: 10.1038/s41467-024-44868-1 (PMC10844649; doi:10.1038/s41467-024-44868-1)
Supplement: Supplementary file 3 — Reporting Summary [file 41467_2024_44868_MOESM3_ESM.pdf]

nature portfolio

Corresponding author(s): Jesmond Dalli
Last updated by author(s): Dec 18, 2023

Reporting Summary

Nature Portfolio wishes to improve the reproducibility of the work that we publish. This form provides structure for consistency and transparency in reporting. For further information on Nature Portfolio policies, see our Editorial Policies and the Editorial Policy Checklist.

Statistics

For all statistical analyses, confirm that the following items are present in the figure legend, table legend, main text, or Methods section.

n/a	Confirmed	
☐	☒	The exact sample size (n) for each experimental group/condition, given as a discrete number and unit of measurement
☐	☒	A statement on whether measurements were taken from distinct samples or whether the same sample was measured repeatedly
☐	☒	The statistical test(s) used AND whether they are one- or two-sided<br>*Only common tests should be described solely by name; describe more complex techniques in the Methods section.*
☐	☒	A description of all covariates tested
☒	☐	A description of any assumptions or corrections, such as tests of normality and adjustment for multiple comparisons
☐	☒	A full description of the statistical parameters including central tendency (e.g. means) or other basic estimates (e.g. regression coefficient) AND variation (e.g. standard deviation) or associated estimates of uncertainty (e.g. confidence intervals)
☐	☒	For null hypothesis testing, the test statistic (e.g. F, t, r) with confidence intervals, effect sizes, degrees of freedom and P value noted<br>*Give P values as exact values whenever suitable.*
☒	☐	For Bayesian analysis, information on the choice of priors and Markov chain Monte Carlo settings
☒	☐	For hierarchical and complex designs, identification of the appropriate level for tests and full reporting of outcomes
☒	☐	Estimates of effect sizes (e.g. Cohen's d, Pearson's r), indicating how they were calculated

Our web collection on statistics for biologists contains articles on many of the points above.

Software and code

Policy information about availability of computer code

Data collection

Lipid mediator data:
Analyst version 1.6.3 and Sciex OS 2.1 were used for the acquisition of LC-MS/MS data.

Flow cytometry data:
FACS Diva version 8.0 was used for the acquisition of flow cytometry data.

FPLC data:
Data for FPLC experiments was acquired using Unicorn V5 software.

Luminescence/Fluorescence data:
e.g. cholesterol efflux assays, free/total cholesterol assays, triglyceride quantification. Data was acquired using Novostar (BMG Labtech) and the associated software (V1.30 R9).

Histology data:
Data for Oil Red O staining of aortas was acquired using the camera built into a Samsung Galaxy A71 mobile phone.
Data for Masson's Trichrome staining of aortic roots was acquired using a Nanozoomer S210 (Hamamatsu).

qPCR data:
Data was acquired using 7900HT Fast Real-Time PCR System (Applied Biosystems) and StepOne System (ThermoFisher).

	Phagocytosis data:<br>Data was acquired using a Celldiscoverer 7 and the ZEN Blue software (Zeiss).
Data analysis	Lipid mediator data were analysed using Sciex OS v2.1, Excel v16.7, Prism v9 and v10, and Metaboanalyst 5.0.<br>Flow cytometry data were analysed using FlowJo (Tree Star Inc) v10, Excel v16.7, Prism v9 and v10, and Metaboanalyst 5.0.<br>FPLC data were analysed using Excel v16.7, and Prism v9 and v10.<br>Luminescence/Fluorescence data were analysed using Excel v16.7, and Prism v9 and v10.<br>Histology data were analysed using ImageJ v1.53, Excel v16.7, and Prism v9 and v10.<br>qPCR data were analysed using Excel v16.7, and Prism v9 and v10.

For manuscripts utilizing custom algorithms or software that are central to the research but not yet described in published literature, software must be made available to editors and reviewers. We strongly encourage code deposition in a community repository (e.g. GitHub). See the Nature Portfolio guidelines for submitting code & software for further information.

Data

Policy information about availability of data

All manuscripts must include a data availability statement. This statement should provide the following information, where applicable:

- Accession codes, unique identifiers, or web links for publicly available datasets
- A description of any restrictions on data availability
- For clinical datasets or third party data, please ensure that the statement adheres to our policy

All data for this study are included in this published article (and its supplementary information files).

Research involving human participants, their data, or biological material

Policy information about studies with human participants or human data. See also policy information about sex, gender (identity/presentation), and sexual orientation and race, ethnicity and racism.

Reporting on sex and gender	The relevance of sex and gender to the observed outcomes was not directly evaluated in the present studies as all the human blood samples we obtained from the NHS blood transfusion services were de-identified. Notably we observed that in experiments performed herein the effects of RvT4 in regulating macrophage response were consistent between donors. Given the likelihood that both male and female donors were included in these studies with a higher likelihood of female donors these results together with those obtained from animal experiments suggests that the biology of RvT4 is retained in both sexes.
Reporting on race, ethnicity, or other socially relevant groupings	The present studies did not evaluate the influence of race and ethnicity on the observed responses
Population characteristics	The study population included healthy individuals who volunteers to donate blood to the NHS Blood Transfusion Services
Recruitment	Participants consented to donate blood for clinical use, and blood cells were collected as by products of the blood collection
Ethics oversight	Ethics approval was provided by the Queen Mary University of London Ethics in Research Committee

Note that full information on the approval of the study protocol must also be provided in the manuscript.

Field-specific reporting

Please select the one below that is the best fit for your research. If you are not sure, read the appropriate sections before making your selection.

☒ Life sciences ☐ Behavioural & social sciences ☐ Ecological, evolutionary & environmental sciences

For a reference copy of the document with all sections, see nature.com/documents/nr-reporting-summary-flat.pdf

Life sciences study design

All studies must disclose on these points even when the disclosure is negative.

Sample size	The number of subjects used in each experiment was determined by carrying out power calculations using historical data presenting strong effect sizes, to reach a power of 80%.
Data exclusions	One sample dataset was excluded from lipid mediator quantification on technical grounds, due to poor sample recovery. One additional sample in a different dataset was excluded from the lipid profiling studies due to poor sample quality.
Replication	Where appropriate experiments were performed on at least two separate occasions.<br>All in vivo experiments repeated were successful.<br>The majority of in vitro experiments repeated were successful, with a small number of experiments excluded based on results obtained from control conditions (ie negative/postiive controls).

Randomization	For in vivo experiments, mice were randomly assigned to different experimental groups.<br>For in vitro studies, cells from individual donors were used in the different experimental groups comparing responses against treatment.
Blinding	For the analysis of in vivo experiments, the experimenter was blinded to group assignment during data collection. The experimenter was also blinded during the earlier stages of data analysis, but were unblinded at stages of analysis that were not ambiguous (eg. taking into account sample recovery or sample mass during lipid mediator analysis).<br><br>For in vitro experiments, the experimenter was not blinded to group assignments during data collection and analysis because since for technical reasons and personnel limitations it was not falsifiable.

Reporting for specific materials, systems and methods

We require information from authors about some types of materials, experimental systems and methods used in many studies. Here, indicate whether each material, system or method listed is relevant to your study. If you are not sure if a list item applies to your research, read the appropriate section before selecting a response.

Materials & experimental systems

n/a	Involved in the study
☐	☒ Antibodies
☐	☒ Eukaryotic cell lines
☒	☐ Palaeontology and archaeology
☐	☒ Animals and other organisms
☒	☐ Clinical data
☒	☐ Dual use research of concern
☒	☐ Plants

Methods

n/a	Involved in the study
☒	☐ ChIP-seq
☐	☒ Flow cytometry
☒	☐ MRI-based neuroimaging

Antibodies

Antibodies used	PE anti-mouse CD64 (FcγRI) Antibody - Biolegend - Cat 139303 - Clone X54-5.71.<br>APC/Cyanine7 anti-mouse F4/80 Antibody - Biolegend - Cat 123117 - Clone BM8.<br>Brilliant Violet 785™ anti-mouse CD11c Antibody - Biolegend - Cat 117335 - Clone N418.<br>Brilliant Violet 421™ anti-mouse LAP (TGF-β1) Antibody - Biolegend - Cat 141407 - Clone TW7-16B4.<br>PE/Cyanine5 anti-mouse/human CD11b Antibody - Biolegend - Cat 101209 - Clone M1/70.<br>iNOS Rabbit mAb (Alexa Fluor® 647 Conjugate) - Cell Signaling Technology - Cat 48866 - Clone D6B6S - Lot 2.<br>TIM-4 Monoclonal Antibody, PerCP-eFluor™ 710 - ThermoFisher Scientific - Cat 46-5866-82 - Clone RMT4-54 - Lot 2609023.<br>PE/Dazzle™ 594 anti-mouse IL-10 Antibody - Biolegend - Cat 505033 - Clone JES5-16E3.<br>APC anti-mouse MERTK (Mer) Antibody - Biolegend - Cat 151507 - Clone 2B10C42.<br>Brilliant Violet 421™ anti-mouse F4/80 Antibody - Biolegend - Cat 123131 - Clone BM8.<br>Alexa Fluor® 647 anti-mouse CD206 (MMR) Antibody - Biolegend - Cat 141711 - Clone C068C2.<br>SR-BI Antibody - Novus Biologicals - Cat NB400-101 - Polyclonal.<br>Goat anti-Rabbit IgG (H+L) Secondary Antibody, FITC - ThermoFisher Scientific - Cat 31635 - Polyclonal - Lot YJ4102801.
Validation	PE anti-mouse CD64 (FcγRI) Antibody:<br>"Each lot of this antibody is quality control tested by immunofluorescent staining with flow cytometric analysis."<br>Ingersoll MA, et al. 2010. Blood 115:e10.<br><br>APC/Cyanine7 anti-mouse F4/80 Antibody:<br>"Each lot of this antibody is quality control tested by immunofluorescent staining with flow cytometric analysis."<br>Rosina M, et al. 2022. Cell Metab. 34:533.<br><br>Brilliant Violet 785™ anti-mouse CD11c Antibody:<br>"Each lot of this antibody is quality control tested by immunofluorescent staining with flow cytometric analysis."<br>Cervantes-Barragan L, et al. 2007. Blood 109:1131.<br><br>Brilliant Violet 421™ anti-mouse LAP (TGF-β1) Antibody:<br>"Each lot of this antibody is quality control tested by immunofluorescent staining with flow cytometric analysis.<br>Oida T, et al. 2010. PLoS One 5:e15523.<br><br>PE/Cyanine5 anti-mouse/human CD11b Antibody:<br>"Each lot of this antibody is quality control tested by immunofluorescent staining with flow cytometric analysis."<br>Charles N, et al. 2010. Nat. Med. 16:701.<br><br>iNOS (D6B6S) Rabbit mAb (Alexa Fluor® 647 Conjugate) Antibody:<br>"...tested in-house for direct flow cytometric analysis in human cells." The product website presents species cross-reactivity with

mouse cells, and a histogram for validation. https://www.cellsignal.com/products/antibody-conjugates/inos-d6b6s-rabbit-mab-alexa-fluor-647-conjugate/48866

TIM-4 Monoclonal Antibody (54 (RMT4-54)), PerCP-eFluor™ 710 Antibody:
"This 54 (RMT4-54) antibody has been tested by flow cytometric analysis of mouse resident peritoneal exudate cells."
doi: 10.1016/j.cell.2021.12.018.

PE/Dazzle™ 594 anti-mouse IL-10 Antibody:
"Each lot of this antibody is quality control tested by intracellular immunofluorescent staining with flow cytometric analysis."
Marques RM, et al. 2021. Cell Death Differ. 28:3140.

APC anti-mouse MERTK (Mer) Antibody:
"Each lot of this antibody is quality control tested by intracellular immunofluorescent staining with flow cytometric analysis."
Zagórska A, et al. 2014. Nat. Immunol. 15:920.

Brilliant Violet 421™ anti-mouse F4/80 Antibody:
"Each lot of this antibody is quality control tested by immunofluorescent staining with flow cytometric analysis."
doi: 10.7554/eLife.70848.

Alexa Fluor® 647 anti-mouse CD206 (MMR) Antibody:
"Each lot of this antibody is quality control tested by intracellular immunofluorescent staining with flow cytometric analysis."
doi: 10.1016/j.immuni.2021.04.025.

SR-BI Antibody - BSA Free:
"We have publications tested in 7 confirmed species: Human, Mouse... We have publications tested in 9 applications: ...FLOW...".
The product website presents flow cytometry results for intracellular staining of SR-BI. https://www.novusbio.com/products/sr-bi-antibody_nb400-101. doi: 10.1161/01.CIR.0000148368.79202.F1.

Goat anti-Rabbit IgG (H+L) Secondary Antibody, FITC:
"Product # 31635 has been successfully used in ...FACS applications."

Eukaryotic cell lines

Policy information about cell lines and Sex and Gender in Research

Cell line source(s)	HL-60 cell line - Supplied by ATCC, Product CCL-240.<br>These cells are promyeoloblasts isolated from the peripheral blood by leukopheresis from a 36-year-old,White, female with acute promyelocytic leukemia.
Authentication	Cell lines were not authenticated since they were procured from ATCC prior to commencing these studies.
Mycoplasma contamination	Cell lines were not tested for mycoplasma contamination.
Commonly misidentified lines<br>(See ICLAC register)	None.

Animals and other research organisms

Policy information about studies involving animals; ARRIVE guidelines recommended for reporting animal research, and Sex and Gender in Research

Laboratory animals	Male, Wild type C57BL/6 of 6-8 weeks were employed for experiments.<br>Male and female ApoE-/- (B6.129P2-Apoe-tm1Unc/J, Jackson Laboratories, stock 002052) mice of 6-8 weeks were employed for experiments.<br>Male and female NOD/Shiltj mice of 10 weeks were donors of K/BxN serum.<br><br>Mice were provided with water and fed ad libitum with either standard laboratory diet or a Western-style diet (Special Diet Services, 829100). Mice were kept on a 12-hour light/dark cycle at an ambient temperature of 20–24°C, at 45-65% humidity.
Wild animals	No wild animals were used in the proposed studies
Reporting on sex	Experiments performed in the present manuscript were primarily conducted using male mice. In the initial study design we had factored using female mice after we had established the mechanisms operative in male mice, however due to the COVID-19 pandemic we had to limit our experiments to male animals since we only had limited access to facilities during this period and were unable to perform the array of studies evaluating the biological activities of RvT4 in female mice. In recent experiments reported in Figure 6F and G we included female mice (2 mice per group) and while the number of mice used is rather small to obtain meaningful conclusions the initial findings suggest that the protective activities of RvT4 are retained in female animals.
Field-collected samples	This study did not involve field collections.

Ethics oversight	Experiments strictly adhered to United Kingdom Home Office regulations (Guidance on the Operation of Animals, Scientific Procedures Act), Laboratory Animal Science Association Guidelines (Guiding Principles on Good Practice for Animal Welfare and Ethical Review Bodies) and according to protocols detailed in a UK Home Office approved protocol (P998AB295).

Note that full information on the approval of the study protocol must also be provided in the manuscript.

Flow Cytometry

Plots

Confirm that:

- [x] The axis labels state the marker and fluorochrome used (e.g. CD4-FITC).
- [x] The axis scales are clearly visible. Include numbers along axes only for bottom left plot of group (a 'group' is an analysis of identical markers).
- [x] All plots are contour plots with outliers or pseudocolor plots.
- [x] A numerical value for number of cells or percentage (with statistics) is provided.

Methodology

Sample preparation	Isolated cells were suspended in DPBS−/− containing 0.02% bovine serum albumin and 1% Fc-blocking IgG (v/v), and incubated with 0.1% LIVE/DEAD Fixable Stain for 20 min on ice. Excess stain was removed and cells incubated with fluorescently-labelled antibodies for 30 min on ice. Cells were washed and then fixed using 1% paraformaldehyde. In select experiments, LipidTOX Green Neutral Lipid Stain (ThermoFisher) was added to cells at 1x, and incubated for 30 minutes. CountBright Absolute Counting Beads were used for leukocyte enumeration. Staining was evaluated using LSRFortessa cell analyzer and analyzed using FlowJo software.
Instrument	BD LSR Fortessa
Software	FlowJo 10 (Tree Star Inc)
Cell population abundance	No sorting was conducted in these studies.
Gating strategy	Doublets were first identified, then live cells were identified using a live/dead stain and the target cell population based on the expression of lineage markers of interest was identified. This is all reported in the Supplement

- [x] Tick this box to confirm that a figure exemplifying the gating strategy is provided in the Supplementary Information.
